# Often Done

**Published:** 1871-10

**Authors:** Joel Benton


### OFTEN DONE.

BY JOEL BENTON.

A little fellow, not supremely wise,
Who had some malady about his eyes—
And suffered pain too tedious to endure—
Sought out a farrier to obtain a cure.

The dull horse-doctor, touched by no remorse,
Served him exactly as he would a horse;
At length, the patient, put in dreadful plight,
Awoke to find his utter loss of sight.

Deeply incensed, the victim—not a saint—
Went to a magistrate with his complaint,
And, throwing on the farrier all the blame,
Demanded damages against his name.

The judge replied, in terms not over-bland,
"In such a case, no damages can stand:
Were not the appellant *an ass*, 'tis sure
He would not seek *a farrier* for a cure."

'Tis sad, indeed, to make a patient blind,
But here the moral is not far to find.

MORAL.

Who takes his maladies to one unwise
Sees little—and, perhaps, may lose his eyes.

